# Association of the Genetic Polymorphisms in Transcription Factor 7-Like 2 and Peroxisome Proliferator-Activated Receptors-***γ***2 with Type 2 Diabetes Mellitus and Its Interaction with Obesity Status in Emirati Population

**DOI:** 10.1155/2015/129695

**Published:** 2015-07-27

**Authors:** Habiba Al-Safar, Ahmed Hassoun, Shaikha Almazrouei, Wala Kamal, Bachar Afandi, Naushad Rais

**Affiliations:** ^1^Department of Biomedical Engineering, Khalifa University of Science, Technology & Research, P.O. Box 127788, Abu Dhabi, UAE; ^2^Dubai Diabetes Centre, Dubai Health Authority, Dubai, UAE; ^3^School of Life Sciences, Manipal University, P.O. Box 345050, Dubai, UAE; ^4^Diabetes Clinics, Tawam Hospital, Al-Ain, UAE

## Abstract

*Background*. Transcription factor 7-like 2 gene (*TCF7L2*) and peroxisome proliferator-activated receptors-*γ*2 (*PPAR-γ2*) have a profound effect on the incidence of type 2 diabetes mellitus (T2DM) and had previously been found to be associated with T2DM risk in various ppopulations. However, studies in the Arab population are inconsistent. We conducted a case control study to confirm the association of variants rs10885409 of *TCF7L2* and *Pro12Ala* (rs1801282) of *PPAR-γ2* with risk of T2DM and related complications in Emirati population of Arab origin. We also investigated the interaction of these associations with obesity status. *Methods*. DNA was extracted from the saliva samples of 272 T2DM patients and 216 nondiabetic Emiratis. Genotyping for rs10885409 (*TCF7L2*) and rs1801282 (*PPAR-γ2 P12A*) variants was accomplished with a TaqMan assay. The subgroups were constituted according to obesity status. *Results*. In the nonobese group, the rs10885409 C allele in the recessive model was significantly associated with the incidence of T2DM (OR 1.975 [95% CI 1.127–3.461], *P* = 0.017), but this association was not observed in the obese group or when BMI was not considered. *PPAR-γ2* risk allele Pro12 frequency (0.96) was similar in the groups tested and more than 90% population was homozygous for this allele. *Conclusions*. Our case-control study is the first of its kind in Emiratis which establishes *TCF7L2* rs10885409 C allele as a T2DM risk factor in Emiratis and this association is modulated by obesity status. We also confirmed that Pro12Ala mutation in *PPAR-γ2* is not associated with T2DM risk in this population.

## 1. Introduction

Insulin resistance in muscle and liver and *β*-cell failure represent the core pathophysiologic defects in type 2 diabetes mellitus (T2DM) [1]. Most patients with T2DM also develop serious complications because of chronic hyperglycemia, such as nephropathy, retinopathy, neuropathy, and cardiovascular diseases, like coronary artery disease, cerebrovascular, and peripheral vascular disease. T2DM involves a complex interaction between genetic variants and the environment, with obesity being a primary risk factor [2]. The correlations between body fat and insulin resistance have been very well established [3], and the associations between genetic polymorphisms and T2DM were demonstrated to be reliant on obesity status [4]. Evidence from various studies has shown that genetic susceptibility to T2DM is polygenic [5, 6], and Genome Wide Association Studies (GWAS) have reported more than 20 genetic loci associated with the risk of T2DM [7, 8]. The single nucleotide polymorphisms (SNPs) in some of these genes have only a small effect on the disease status, but few, like those at the transcription factor 7-like 2 gene (*TCF7L2*) and proliferator-activated receptors-*γ*2 (*PPAR-γ2*) have a profound effect on the T2DM prevalence, as has previously been reported in large scale studies or meta-analyses conducted in various populations and ethnic groups [9–11].

*TCF7L2* variant rs7903146 a C-to-T (genomic position: 114748339) substitution in intron 3 and* PPAR-γ2 Pro12Ala* have been most extensively studied in all major ethnic groups and were found to be more consistently associated with the risk of developing T2DM in most of populations, such as Asians, Africans, and Caucasians [12, 13]. These associations were also observed in most of the Arab populations of MENA region like Tunisians, Lebanese, [14–18], Iranian [19, 20], and North Africans [21]. Although these polymorphisms were established as risk factor for T2DM in most of the populations studied so far, however, some of the studied polymorphisms seem not to be a major contributor to T2DM susceptibility in the Saudi and Emirati Arab populations [22–24]. There is considerable inter-population variation in the frequency of the risk allele (*Pro12*) which ranges from a high of 0.96–0.98 in populations including the Japanese, Chinese, and African Americans to 0.91 in Pima Indians and a low of 0.81 in the Finnish population [25].* TCF7L2* is an important regulator of the proglucagon gene, which encodes* GLP-1* and is believed to play a role in glucose metabolism. Evidence also suggests that this transcription factor itself is critical for beta cell proliferation, antiapoptotic activity, and insulin secretion [26, 27]. GWAS also identified the* TCF7L2* genes to be associated with T2DM risk [28]. Moreover, in some studies the associations of SNPs in* PPAR-γ2* and* TCF7L2* have previously been shown to be modulated by obesity status [4].* PPAR-γ2 Pro12Ala* polymorphism was found more associated with T2DM susceptibility in obese populations while* TCF7L2* rs7903146 T allele was shown to be more prevalent in T2DM nonobese population compared to T2DM obese individuals, suggesting a pivotal role of obesity status in genetic association with T2DM [4]. Therefore, inconsistence regarding the genetic association studies among the Arab populations might be partly explained by the obesity status.

Based on these findings, we decided to study the effect of obesity status on association of* PPAR-γ2 Pro12Ala* variant with T2DM risk in Emirati population. In addition, we have also chosen to evaluate genetic variant rs10885409 in* TCF7L2* which is C114798062T substitution and was reportedly associated with T2DM risk in North Indians [5] but has not been examined in earlier genetic epidemiologic studies in any other previously studied populations, including Arabs. We also studied the effect of these variants on diabetic complications, which had not been previously reported in any of the Arab populations studied to date.

## 2. Methodology

### 2.1. Subjects and Sample Collection

This study was conducted on four hundred and eighty-six (*n* = 486) unrelated Emiratis who were identified during their routine visit to a diabetes clinic in Dubai and Al Ain, United Arab Emirates, between the period of June 2012 and December 2013. The case group of 272 Type 2 Diabetes patients had mean age of 58 ± 12 years and consisted of 60% females. The control group consisted of 216 healthy individuals who had mean age 45 ± 16 years and was 68.37% female. The individuals selected for control group were nondiabetic, normotensive individuals with no indication of any complications. All participants gave their informed consent in writing. This study was approved by the Ethics Committees of the Dubai Health Authority and Al Ain Medical District Human Research Ethics Committee in the United Arab Emirates.

After obtaining an informed consent from the participant, one mL of saliva was collected by a registered nurse using Oragene kit OGR-500 (DNA Genotek, Ottwa, Canada). The genomic DNA was extracted from saliva using prepITTM•L2P (DNA Genotek, Ottwa, Canada) in accordance with the manufacturer's instructions. All the saliva samples were processed in the Molecular Cell Biology laboratory at Khalifa University, Abu Dhabi, for DNA extraction and genotyping. In addition, a clinical assessments and lifestyle questionnaire was completed at the clinic to study any correlation between lifestyle variables and genetic variation. An individual was classified as T2DM if the subject [1] was diagnosed with T2DM by a qualified physician, [2] was on a prescribed drug treatment regimen for T2DM, and [3] returned biochemical test results of a fasting plasma glucose level of at least 126 mg/dL based on the criteria outlined by the World Health Organization (WHO) consultation group report. The individuals with BMI more than 30 were considered obese and those with a BMI less than 30 were grouped into the nonobese population. According to JNC 8 classification, all the individuals with blood pressure more than 140/90 mmHg were considered hypertensive.

### 2.2. Genotyping for* TCF7L2* rs10885409 and* PPAR-γ2 Pro12Ala* Variants

TaqMan assays were used for SNP genotyping (Applied Biosystems, Foster City, CA). These assays use fluorogenic probes in a 5′ nuclease assay to identify differences in DNA sequences. The laboratory-designed probes were obtained from Applied Biosystems. All PCR reactions took place in optical 96-well reaction plates (Applied Biosystems) with a final reaction volume of 10 *μ*L that contained 10 ng of genomic DNA, 5 *μ*L of TaqMan Genotyping Master Mix (Applied Biosystems), and 0.5 *μ*L of assay mix (20x). The PCR thermal cycling conditions were set as follows: 95°C for 10 min to activate DNA polymerase, followed by 40 cycles of 92°C for 15 s and 60°C for 1 min. All PCRs were performed using a 9700 fast thermal cycler (Applied Biosystems), and the end point fluorescence readings were obtained on a ViiA 7 Real-time PCR system (Applied Biosystems). Upon running 5% samples in duplicate, the genotyping success rates were found to be 99.9% for both SNPs. Negative control was used in each run for quality control purposes.

### 2.3. Statistical Methods

Statistical analyses were performed using STATA version 13 (STATA Corp., TX, USA). Student's *t*-test was used to compare the clinical parameters to different groups. The genotype frequencies were tested for the Hardy-Weinberg equilibrium using a *χ* ^2^ test. Fisher Exact test was used to analyze the statistical significance of the difference in allelic distribution of various polymorphisms in patients and controls, with *P* values, odds ratios (ORs), and confidence intervals reported. The following gene transmission models were considered for* TCF7L2* to study the effect of T allele: (1) recessive (TTVs CT + CC) and (2) dominant (CC Vs CT + TT) effect of T allele for association analysis. A *P* value of < 0.05 was considered statistically significant.

## 3. Results

The demographic characteristics and clinical and biochemical parameters of T2DM patients and nondiabetic control patients are described in Table 1. The mean age and male/female ratios significantly differed in the affected individuals and controls (Table 1). The mean glucose level, mean arterial blood pressure, triglycerides, LDL, and total cholesterol by* TCF7L2* rs10885409 genotype did not significantly differ (CC Vs CT + TT) (Table 2). The allele and genotype frequencies of the rs10885409 SNP at* TCF7L2* and *PPAR-γ2* *Pro12Ala* variant in control and T2DM groups without consideration of obesity status are summarized in Table 3. The distribution of the* TCF7L2* polymorphism (rs10885409) was consistent with the Hardy-Weinberg equilibrium in T2DM patients (*χ* ^2^ = 2.513, *P* = 0.2846) as well as in nondiabetic controls (*χ* ^2^ = 1.296, *P* = 0.5230). The frequencies of TT, CT, and CC genotypes for rs10885409 SNP at* TCF7L2* were 22.5, 44.6, and 32.9%, respectively, in T2DM cases and 23.3, 46.5, and 30.2%, respectively, in nondiabetic subjects. T allele was found with minor allele frequencies of 0.45 and 0.48 in T2DM cases and control groups, respectively. The genotype and allele frequencies were not statistically different between the groups. The genotyping results for* PPAR-γ2* demonstrated G allele (12Ala) as a minor allele with a frequency of 0.04 and which was found only in heterozygote form. The genotype and allele frequencies were almost similar in T2DM cases and controls. Deviations from the Hardy-Weinberg equilibrium were not observed in any of the groups (*χ* ^2^ = 0.669, *P* = 0.7156 for control group).

### 3.1. Association of the* TCF7L2* rs10885409 Variant with the Risk of T2DM and Related Complications in a Case-Control Study 

#### 3.1.1. All Control and T2DM Individuals

Association analyses demonstrated no significant association between the rs10885409 SNP of* TCF7L2* and T2DM susceptibility when the obesity status was not considered (Tables 3 and 4). Moreover, the genotype and allele frequency distributions among the T2DM case groups with or without hypertension or any of the complications related with T2DM did not significantly differ (Table 6).

#### 3.1.2. Nonobese T2DM Association

When the data were analyzed considering the obesity status, the frequency of the CC genotype was found to be significantly higher (26.1 Vs 41.1, *P* = 0.0176) in nonobese (BMI < 30) T2DM patients, but not in the obese affected patients (BMI > 30) (Table 5). The nonobese subjects with CC genotypes were at an approximately two-fold higher risk of T2DM than those with CT or TT genotypes (OR 1.975 (95% CI 1.127–3.461), *P* = 0.017) (Table 5).

## 4. Discussion

In this investigation, we carried out genetic association study of the SNPs in* PPAR-γ2* and* TCF7L2* with T2DM susceptibility and its interaction with the obesity status for the first time in the Emirati population. While many variants have been identified in* PPAR-γ2* gene, the most prevalent and best studied is the* Pro12Ala* polymorphism which has been shown to impair the function of the PPAR-*γ*2 isoform of the receptor and to be associated with obesity and/or diabetes and insulin sensitivity related phenotypes in different populations [9, 12, 13]. Grant et al. [29] found that common genetic variants of the* TCF7L2* gene were associated with T2DM risk, and these findings were consistently reproduced in several populations. Most of these studies focused on the genetic variant rs7903146, which was consistently found to be associated with a risk for T2DM in most populations studied to date. However, the scenario in the Arab population was a slightly different. Studies of two major Arab populations, Saudi Arabs and Emirati Arabs [22, 23], were not consistent with these findings. Saadi et al. [23] and Alsmadi et al. [22] already examined the variant s7903146 in Emirati and Saudi populations, respectively. We did not imitate their reports. Rather, we investigated another SNP rs10885409 that was found to be associated with T2DM in a previous study conducted in Indians Sikhs residing in the USA [9].

The genotyping results for* PPAR-γ2* showed* 12Ala* allele as minor allele and we established the risk allele (*Pro12*) frequency in Emirati population among the highest observed so far and was comparable to Saudi [24], Japanese, Chinese, and African but failed to observe any association with T2DM risk. These findings are consistent with Saudi [24] and some Caucasian studies [30, 31]. Moreover, given the very high incidence of the* Pro12* allele in this population, the study size was extremely underpowered and the data could not be analysed for the association with incidence of T2DM related complications and interaction with obesity status.

The results of the genotyping for rs10885409 demonstrated that neither the T nor the C allele was associated with T2DM. Similarly, rs10885409 did not correlate with T2DM when the data for males and females were analyzed separately, ruling out the possibility of any gender-based association. We also observed that the genotype did not affect any of the clinical or biochemical parameters, such as the BMI, fasting glucose level, or lipid profile (Table 2), which are considered to be related to T2DM and were found significantly altered in the cases and controls (Table 1). The allele and genotype distribution were not in concordance with the only available study conducted in an Indian Sikh population on the same allele [9]. The C allele was established to be minor, with an allele frequency of 0.46 in nondiabetics and significantly higher frequency of 0.53 in T2DM cases, and the strongest association was suggested (1.64; 95% CI [1.20–2.24]; *P* = 0.001) in dominant models [9]. The data presented here and in the two previous studies in Arabs [16, 17] suggested that* TCF7L2* polymorphism is not related to T2DM risk in the Arabian Peninsula. A coding variant (*Pro477Thr*) in exon 14 of* TCF7L2* and the recently identified rs290487 variant of* TCF7L2* were also not associated with T2DM in Japanese [32] and Chinese [33] populations. However, rs7903146 was associated with T2DM in both of these populations [9, 10, 30], suggesting the possibility of allele-specific associations only. A literature exploration revealed inconsistencies in the reports in even the most thoroughly studied and widely accepted T2DM risk variant rs7903146, especially in Arab populations. In Arabian Peninsula, the life style is more westernized with reduced physical activity and excessive calorie intake. These divergent results of the association could be explained by the interaction between the BMI and environmental factors in modulating T2DM risk, as previous reports suggested that different genetic architectures could increase the T2DM susceptibility according to the obesity status [4]. In fact, the TCF7L2 rs7903146 T allele was associated with T2DM in nonobese Tunisian Arabs, whereas no effect was detected in overweight and obese individuals [31]. We analyzed our results while considering obesity. We divided our T2DM cases and controls into two groups each of BMI < 30 and BMI > 30. The obese group included 147 T2DM and 88 healthy subjects, and the nonobese group included 112 and 115 T2DM and control individuals, respectively. We observed the frequency of the CC genotype to be significantly higher (41.1 versus 26.1, *P* = 0.0176) in nonobese T2DM cases compared to matching nondiabetic subjects (0.61 versus 0.51, *P* = 0.0376), and the same was also true for the C allele (Table 4). The nonobese CC genotype carriers were found to be at high risk for T2DM (OR 1.975, 95% CI 1.127–3.461, *P* = 0.017) (Table 5); however, rs10885409 was not associated with T2DM risk in the obese group. These results were in complete concordance with those of the Tunisian study [20] and were sufficient to explain the inconsistencies that were observed regarding the effect of the* TCF7L2* variant on T2DM risk. Most of the studies were conducted without considering the obesity status, and our results strongly suggested that the genetic associations might be modulated by the presence and absence of obesity, especially in Arab populations.

The allelic and genotypic associations were also analyzed in diabetic patients with at least one complication and without any complications. We did not observe any significant differences in the allele and genotype frequencies between the two groups (Table 6), indicating that rs10885409 did not affect the presence of diabetes-related complications. Studies regarding the effect of variant rs10885409 on diabetes-related complications were not available, and the studies on rs7903146 were largely inconsistent [34, 35]. Unfortunately, we could not analyze this association for each complication separately or based on obesity status because of the very small sample size of the groups formed for this purpose. However, we observed significant differences in the biochemical parameters in different genotypes in nonobese subjects. The mean levels of cholesterol, LDL, and TGs were significantly higher in T2DM cases with either CT or TT genotypes compared to matching controls with the same genotype (Table 7). Interestingly, the LDL levels were significantly higher in T2DM subjects with the CT + TT genotype compared to T2DM cases with the CC genotypes (*P* = 0.0001) (Table 7).

In conclusion, our results show that the* Pro12Ala* mutation in the* PPAR-γ2* gene is unlikely to serve as clinically useful predictor of T2DM and/or obesity in Emiratis.* TCF7L2* variation may be a risk factor for the occurrence of T2DM in Arab populations, but this association relies on the obesity status. Our case control study on rs10885409 established CC genotype as a risk factor in nonobese Emiratis. However, the association was absent when the obesity status was not considered and in the obese population with T2DM risk and related complications.

## Figures and Tables

**Table 1:** Clinical and biochemical characteristics of T2DM patients and healthy subjects.

	T2DM patients *n* = 272	Nondiabetic subjects *n* = 216	*P* value
Age (years)	58 ± 12	45 ± 16	<0.0001
Male : female ratio	110 : 162	69 : 147	0.05
BMI	31.99 ± 6.3	29.97 ± 6.14	0.0009
Fasting glucose (mmol/L)	8.99 ± 7.23	5.63 ± 7.23	<0.0001
Systolic blood pressure	129.97 ± 17.24	122.50 ± 15.65	<0.0001
Diastolic blood pressure	70.0 ± 11.0	71.35 ± 11.45	0.6197
HB1AC	7.6 ± 1.6	5.59 ± 0.56	<0.0001
Cholesterol (mmol/L)	4.14 ± 1.05	4.49 ± 0.96	0.0001
Triglycerides (mmol/L)	1.46 ± 0.83	1.18 ± 0.76	0.0041
HDL (mmol/L)	1.22 ± 0.49	1.31 ± 0.51	0.1316
LDL (mmol/L)	2.30 ± 0.91	2.66 ± 0.93	0.0008

**Table 2:** Clinical and biochemical characteristics in different genotypes.

	CC *n* = 272	TT + CT *n* = 216	*P* value
BMI	31.21 ± 5.6	31.15 ± 6.4	0.9399
Fasting glucose (mmol/L)	7.41 ± 3.15	7.42 ± 3.15	0.9829
Systolic blood pressure	128.68 ± 18.0	126.38 ± 16.71	0.2374
Diastolic blood pressure	71.76 ± 11.06	70.79 ± 11.29	0.4551
HB1AC	6.94 ± 1.64	7.36 ± 3.54	0.2427
Cholesterol (mmol/L)	4.18 ± 1.00	4.31 ± 1.06	0.2667
Triglycerides (mmol/L)	1.36 ± 0.87	1.37 ± 0.83	0.9144
HDL (mmol/L)	1.25 ± 0.37	1.25 ± 0.56	0.8946
LDL (mmol/L)	2.31 ± 0.87	2.49 ± 0.96	0.0888

**Table 3:** Genotype and allele frequency distribution of rs10885409 of TCF7L2 and PPAR-*γ* P12A mutation without obesity consideration.

	Total *N*	T2DM patients *N* (%)	Nondiabetic subjects *N* (%)	*P* value
*TCF7L2* genotypes				
TT	111	61 (22.5)	50 (23.3)	NS
CT	221	121 (44.6)	100 (46.5)	NS
CC	154	89 (32.9)	65 (30.2)	NS
Allele frequencies				
T	443	243 (0.45)	205 (0.48)	NS
C	529	299 (0.55)	225 (0.52)	NS
PPAR-*γ* genotypes				
CC	447	250 (91.9)	197 (91.2)	NS
CG	41	22 (8.1)	19 (8.8)	NS
GG	0	0	0	NS
Allele frequencies				
C	935	522 (0.96)	413 (0.96)	NS
G	41	22 (0.04)	19 (0.04)	NS

**Table 4:** Genotype and allele frequency distribution for *TCF7L2* rs10885409 variant and the risk of type 2 diabetes in the Emirati population in various groups based on obesity status.

	Without consideration of BMI			Obese (BMI >30)			Nonobese (BMI <30)		
	T2DM	Healthy	*P *	T2DM	Nondiabetics	*P *	T2DM	Nondiabetics	*P *
Genotypes									
CC	89 (32.8%)	65 (30.2%)	0.557	42 (28.2%)	31 (35.2%)	0.3082	46 (41.1%)	30 (26.1%)	0.0176
CT + TT	182 (67.2%)	150 (69.8%)		107 (71.8%)	57 (64.8%)		66 (58.9%)	85 (73.9%)	

Allele frequencies									
C	0.55	0.52	0.399	0.52	0.56	0.342	0.61	0.51	0.0376
T	0.45	0.48		0.48	0.44		0.39	0.49	

**Table 5:** Analysis of association of *TCF7L2* rs10885409 variant and T2DM in case-control study by obesity status.

Model	Without consideration of BMI			Obese (BMI >30)			Nonobese (BMI <30)		
	OR	95% CI	*P* value	OR	95% CI	*P* value	OR	95% CI	*P* value
CC Vs CT + TT	1.128	0.767–1.660	0.539	0.722	0.410–1.269	0.258	1.975	1.127–3.461	0.017
TT Vs CT + CC	0.927	0.598–1.438	0.736	1.083	0.580–2.021	0.802	0.752	0.396–1.428	0.384

Allele frequencies									
C Vs T	1.1232	0.8714–1.4477	0.37	0.843	0.580–1.226	0.372	1.495	1.029–2.170	0.035

**Table 6:** Analysis of association of *TCF7L2* rs10885409 variant and T2DM related complications in Emirati population in case-control study.

	Total *N* (*n* = 232)	With complications *N* = 105 (%)	Without complications *N* = 127 (%)	*P* value
*TCF7L2* genotypes				
TT	48	26 (4.8)	22 (17.3)	0.3135^∗^
CT	115	44 (41.9)	71 (55.9)	
CC	69	35 (33.3)	34 (26.8)	

Allele frequencies				
T	211	96 (0.46)	115 (0.45)	0.9257
C	253	114 (0.54)	139 (0.55)	

^∗^CC Vs CT + TT.

**Table 7:** Biochemical parameters in different *TCF7L2* rs10885409 genotypes in nonobese Emirati T2DM cases and controls.

Genotypes	Cholesterol (mmol/L)		*P *	LDL (mmol/L)		*P *	HDL (mmol/L)		*P *	TGS (mmol/L)		*P *	HB1Ac (mmol/L)		*P *
	T2DM	Nondiabetics		T2DM	Nondiabetics		T2DM	Nondiabetics		T2DM	Nondiabetics		T2DM	Nondiabetics	
CC	4.12 ± 1.0	4.56 ± 0.68	0.1805	2.28 ± 0.94	2.57 ± 0.81	0.2700	1.3 ± 0.39	1.28 ± 0.39	0.8597	1.34 ± 0.84	1.37 ± 1.26	0.9157	7.62 ± 1.87	5.48 ± 0.44	**0.0003**
CT + TT	4.05 ± 0.83	4.55 ± 1.13	**0.0175**	3.86 ± 0.76	2.55 ± 0.98	**0.0001**	1.28 ± 0.84	1.42 ± 0.71	0.4050	1.4 ± 0.81	1.04 ± 0.44	**0.0221**	7.64 ± 1.96	5.76 ± 0.73	**0.0001**
*P* values	0.7565	0.9718		0.0001	0.9393		0.8946	0.4379		0.7375	0.1755		0.9611	0.2302	
